# Cumulative score based on preoperative plasma fibrinogen and serum C-reactive protein could predict long-term survival for esophageal squamous cell carcinoma

**DOI:** 10.18632/oncotarget.11145

**Published:** 2016-08-09

**Authors:** Rui Tian, Hong Yan, Fei Zhang, Peng Sun, Ai-Ran Wu, Min Zhang, Yu-Lu Jiang, Jing Wu, Yan-Hong Lu, Qiu-Yan Xu, Xiao-Hong Zhan, Rong-Xin Zhang, Li-Ting Qian, Jie He

**Affiliations:** ^1^ Department of Pathology, Anhui Cancer Hospital & Anhui Provincial Hospital Affiliated Anhui Medical University, Hefei, Anhui, P. R. China; ^2^ Collaborative Innovation Center for Cancer Medicine, Guangzhou, Guangdong, P. R. China; ^3^ State Key Laboratory of Oncology in South China, Guangzhou, Guangdong, P. R. China; ^4^ Department of Medical Oncology, Sun Yat-sen University Cancer Center, Guangzhou, Guangdong, P. R. China; ^5^ Department of Thoracic Surgery, Anhui Cancer Hospital & Anhui Provincial Hospital Affiliated Anhui Medical University, Hefei, Anhui, P. R. China; ^6^ Department of Radiology, Anhui Provincial Hospital & Anhui Provincial Hospital Affiliated Anhui Medical University, Hefei, Anhui, P. R. China

**Keywords:** esophageal squamous cell carcinoma, fibrinogen, C-reactive protein, survival

## Abstract

The present study was to establish a prognostic indicator based on preoperative fibrinogen and C-reactive protein (CRP) (FC score) in esophageal squamous cell carcinoma (ESCC). Clinicopathologic characteristics, preoperative plasma fibrinogen and serum CRP levels were reviewed in patients who underwent transthoracic esophagectomy. The optimal cut-off value for fibrinogen and CRP was defined as 4.0 g/dL and 10.0 mg/L according to previous reports. Patients with elevated fibrinogen and CRP levels were assigned a score of 2, those with only one of these two abnormalities were allocated a score of 1, and those with neither of the two abnormalities were assigned a score of 0. Preoperative FC score was significantly correlated with degree of differentiation, depth of invasion, tumor-node-metastasis (TNM) stage and modified Glasgow Prognostic Score (mGPS). No significant differences in age, gender, tumor length, tumor location, lymph node status or smoking were identified between groups. Univariate survival analysis demonstrated that high preoperative FC score (1/2) was significantly associated with impaired disease free survival (DFS) [hazard ratio (HR), 1.650; 95% confidence interval (CI), 1.181-2.303; *P* = 0.003] and overall survival (OS) (HR, 1.879; 95% CI, 1.333-2.648; *P*<0.001), and it remained an independent predictor for both DFS (HR, 1.468; 95% CI, 1.043-2.067; *P*=0.028) and OS (HR, 2.070; 95% CI, 1.266-3.385; *P*=0.004) in multivariate Cox regression analysis. Preoperative FC score might represent a new potential marker of worst prognosis that warrants further evaluation in prospective and large cohort studies among ESCC patients who underwent transthoracic esophagectomy.

## INTRODUCTION

Esophageal cancer is the sixth most common cancer and fourth leading cause of cancer-related death in China [1]. In contrast to Western countries, esophageal squamous cell carcinoma (ESCC) continues to be the predominant subtype in Chinese population, with a high burden of morbidity and mortality [1–3]. Although great progress has been made in the diagnosis and treatment of ESCC in last decades, the prognosis remains unfavorable, with a 5-year overall survival (OS) rate of less than 40% [1].

The American Joint Committee on Cancer (AJCC), the Union for International Cancer Control (UICC) tumor-node-metastasis (TNM) staging system, and the histopathological findings are the most widely used prognostic factors to stratify survival in ESCC [4–6]. Besides, some inflammation-based prognostic indexes, such as the Glasgow Prognostic Score (GPS), modified GPS (mGPS), the preoperative plasmatic gamma-glutamyltransferase levels, new data regarding platelet-lymphocyte ratio (PLR), neutrophil-lymphocyte ratio (NLR) and lymphocyte-monocyte ratio (LMR) have also been identified as potential prognostic factors in ESCC [7–11]. However, more accurate indicators are still desirable for risk classification and optimal management of ESCC patients.

Rather than serving as an indicator of activated hemostatic system and a trigger for increased thromboembolic events, hyperfibrinogenemia is frequently observed in various malignancies, including ESCC, and has been demonstrated to play vital roles in tumor progression, invasion and distant metastasis [12–15]. In addition, it has also been identified to be significantly associated with advanced clinical stage and unfavorable survival in ESCC patients [16–20]. Furthermore, systemic inflammation has been reported to be associated with impaired survival in patients with cancer [21–22]. However, inflammation and C-reactive protein (CRP) levels are not always elevated in esophageal cancer patients [23–24], leaving CRP insufficient for high-risk stratification. Therefore, we proposed that in combination with fibrinogen, CRP might provide a more accurate prediction formula in predicting long-term survival for resectable ESCC patients.

The purpose of this present study was to assess the relationship between preoperative fibrinogen and CRP (FC) score and clinicopathologic variables, and to investigate its prognostic significance in ESCC patients.

## RESULTS

### Patient characteristics

The baseline clinicopathologic characteristics of the included 260 patients were demonstrated in Table 1. The median age at diagnosis was 59.0 years (ranged, 20.0-87.0 years). The vast majority (74.2%) of the patients were males. Primary tumors were located at the middle esophagus in 61.5 % of the patients. And the numbers of patients with poorly/not differentiated, moderately differentiated and well differentiated tumors were 58 (22.3%), 135 (51.9%) and 67 (25.8%) respectively. Tumor invasion depth of T1, T2, T3, and T4 were observed in 25 (9.6%), 46 (17.7%), 168 (64.6%), and 21 (8.1%) of the patients, respectively. Lymph node metastasis was negative in 144 (55.0%) of the patients. Of these, 24 (9.2%) were stage I, 127 (48.8%) were stage II and 107 (41.9%) were stage III (Table 1).

**Table 1:** Correlation between preoperative FC scores and clinicopathological characteristics in 260 ESCC patients

Clinicopathologic characteristics	Patients N (%)	FC score (N, %)			*P* value
		0	1	2	
Age (years)					0.962
< 60	150 (57.7)	106 (57.3)	26 (57.8)	18 (60.0)	
≥ 60	110 (42.3)	79 (42.7)	19 (42.2)	12 (40.0)	
Gender					0.224
Male	193 (74.2)	133 (71.9)	38 (84.4)	22 (73.3)	
Female	67 (25.8)	52 (28.1)	7 (15.6)	8 (26.7)	
Tumor location					0.177
Upper	20 (7.7)	14 (7.6)	4 (8.9)	2 (6.7)	
Middle	160 (61.5)	120 (64.9)	27 (60.0)	13 (43.3)	
Lower	80 (30.8)	51 (27.5)	14 (31.1)	15 (50.0)	
Tumor length (cm)					0.055
< 5	149 (57.3)	114 (61.6)	23 (51.1)	12 (40.0)	
≥ 5	111 (42.7)	71 (38.4)	22 (48.9)	18 (60.0)	
Differentiation					0.043*
Well	67 (25.8)	50 (27.1)	5 (11.1)	12 (40.0)	
Moderate	135 (51.9)	92 (49.7)	28 (62.2)	15 (50.0)	
Poor/Undifferentiated	58 (22.3)	43 (23.2)	12 (26.7)	3 (10.0)	
T stage					0.007*
T1	25 (9.6)	24 (13.0)	0	1 (3.3)	
T2	46 (17.7)	36 (19.5)	6 (13.3)	4 (13.4)	
T3	168 (64.6)	112 (60.5)	37 (82.3)	19 (63.3)	
T4	21 (8.1)	13 (7.0)	2 (4.4)	6 (20.0)	
N stage					0.698
N0	148 (55.8)	104 (56.3)	23 (51.1)	16 (53.3)	
N1	67 (25.3)	50 (27.0)	9 (20.0)	8 (26.7)	
N2	40 (15.1)	25 (13.5)	10 (22.2)	5 (16.7)	
N3	10 (3.8)	6 (3.2)	3 (6.7)	1 (3.3)	
TNM stage					0.048*
I	24 (9.2)	23 (12.4)	0	1 (3.3)	
II	127 (48.8)	91 (49.2)	23 (51.1)	13 (43.3)	
III	109 (41.9)	71 (38.4)	22 (48.9)	16 (53.4)	
Smoking					0.495
Never	100 (38.5)	75 (40.5)	14 (31.1)	11 (36.7)	
Ever	160 (61.5)	110 (59.5)	31 (68.9)	19 (63.3)	
Alcohol consumption					0.071
Never	170 (65.4)	128 (69.2)	23 (51.1)	19 (63.3)	
Ever	90 (34.6)	57 (30.8)	22 (48.9)	11 (36.7)	
mGPS					< 0.001*
0	213 (81.9)	185 (100.0)	28 (62.2)	0	
1	42 (16.2)	0	13 (28.9)	29 (96.7)	
2	5 (1.9)	0	4 (8.9)	1 (3.3)	

FC, fibrinogen and C-reactive protein; ESCC, esophageal squamous cell carcinoma; TNM, tumor-node-metastasis; mGPS, modified Glasgow prognostic score.

* *P* < 0.05.

### Correlation between preoperative FC score and clinicopathologic parameters

Among the 260 patients, 185 (71.2 %) had an FC score of 0 and 75 (28.8%) had an FC score of 1 or 2 (Table 1). The results demonstrated that preoperative FC score was significantly correlated with degree of differentiation, depth of invasion, tumor-node-metastasis (TNM) stage and mGPS. Whereas no significant differences in age, gender, tumor length, tumor location, lymph node status or smoking were identified between groups (Table 1). Notably, an mGPS of 1 or 2 was significantly observed more frequently among patients with high preoperative FC score (Table 1).

### Prognostic value of preoperative FC score in predicting long-term survival for ESCC

The median follow-up time was 46.5 months. There were one hundred and forty-one patients died during the follow-up period, with an estimated median DFS and OS of 35.5 months (95%CI, 19.0-52.0 months) and 56.3 months (95%CI, 38.1-74.5 months), respectively.

**Figure 1:**
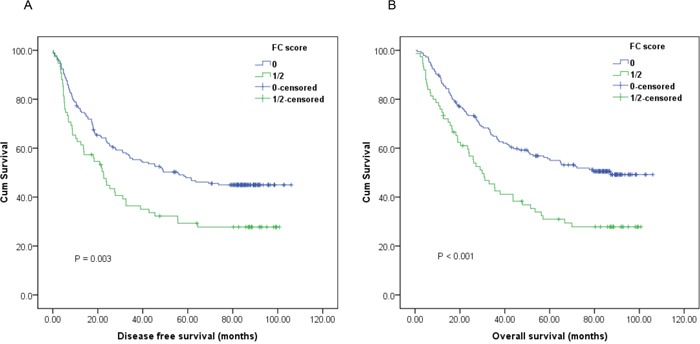
Kaplan-Meier survival curves of **A.**, disease-free survival (DFS) and **B.**, overall survival (OS) stratified by preoperative fibrinogen and C-reactive protein (FC) score in 260 esophageal squamous cell carcinoma (ESCC) patients (with log-rank test).

A Cox univariate model for DFS showed that high preoperative FC score was significantly associated with unfavorable DFS (HR, 1.650; 95%CI, 1.181-2.303; *P*=0.003; Figure 1A). Gender (Male/Female), tumor length (<5/≥5 cm), depth of invasion (T1-2/T3-4), lymph node involvement (Negative/Positive), TNM stage (I-II/III), smoking (Never/Ever) and alcohol consumption (Never/Ever) were other significant prognostic variables identified by univariate analysis (*P*<0.05). On multivariate analysis, TNM stage (HR, 2.209; 95% CI, 1.577-3.095; *P*<0.001), smoking (HR, 1.755; 95% CI, 1.047-2.942; *P*=0.033) and preoperative FC score (HR, 1.468; 95% CI, 1.043-2.067; *P*=0.028) were suggested to be independent prognostic factors for DFS (Table 2).

**Table 2:** Univariate and multivariate analysis of DFS in 260 ESCC patients

Variables	Univariate			Multivariate		
	HR	95% CI	*P*	HR	95% CI	*P*
Age (years)						
≥ 60	1		0.940			NI
< 60	1.012	0.736-1.393				
Gender						
Male	1		0.044*	1		0.554
Female	0.672	0.457-0.989		1.182	0.679-2.056	
Tumor location						
Lower	1		0.894			NI
Middle	0.929	0.654-1.318				
Upper	1.019	0.541-1.922				
Tumor length (cm)						
< 5	1		0.019*	1		0.203
≥ 5	1.464	1.066-2.012		1.179	0.848-1.640	
Differentiation						
Well/Moderate	1		0.788			NI
Poor/Undifferentiated	1.053	0.724-1.530				
Depth of invasion						
T1/T2	1		< 0.001*			NI
T3/T4	2.147	1.438-3.205				
Lymph node involvement						
Negative	1		< 0.001*			NI
Positive	2.854	2.056-3.962				
TNM stage						
I/II	1		< 0.001*	1		< 0.001*
III	2.439	1.766-3.368		2.209	1.577-3.095	
Smoking						
Never	1		0.002*	1		0.033*
Ever	1.734	1.229-2.448		1.755	1.047-2.942	
Alcohol consumption						
Never	1		0.019*	1		0.758
Ever	1.476	1.065-2.044		0.941	0.638-1.387	
mGPS						
0	1		0.123			NI
1/2	1.360	0.920-2.008				
FC score						
0	1		0.003*	1		0.028*
1/2	1.650	1.181-2.303		1.468	1.043-2.067	

DFS, disease-free survival; HR, hazard ratio; CI, confidence interval; NI, not included.

* *P* < 0.05.

Univariate analysis of OS indicated that patients with high preoperative FC score tended to have impaired OS (HR, 1.879; 95% CI, 1.333-2.648; *P*<0.001; Figure 1B). Besides, other parameters including depth of invasion, lymph node involvement, TNM stage, smoking, alcohol consumption and mGPS could also significantly predict OS. Multivariate analysis was then performed using a Cox proportional hazards model. After adjusting for other confounding variables, we found that high FC score could also serve as an independent predictor for OS (HR, 2.070; 95% CI, 1.266-3.385; *P*=0.004). As expected, TNM stage was another significant predictor for OS. Compared with TNM stage I-II patients, those with TNM stage III had poorer OS (HR, 2.150; 95% CI, 1.523-3.034; *P*<0.001) (Table 3).

**Table 3:** Univariate and multivariate analysis of OS in 260 ESCC patients

Variables	Univariate			Multivariate		
	HR	95% CI	*P*	HR	95% CI	*P*
Age (years)						
≥ 60	1		0.450			NI
< 60	1.136	0.816-1.582				
Gender						
Male	1		0.060			NI
Female	0.681	0.457-1.016				
Tumor location						
Lower	1		0.876			NI
Middle	0.933	0.646-1.368				
Upper	1.069	0.550-2.076				
Tumor length (cm)						
< 5	1		0.081			NI
≥ 5	1.344	0.965-1.873				
Differentiation						
Well/Moderate	1		0.791			NI
Poor/Undifferentiated	1.054	0.716-1.550				
Depth of invasion						
T1/T2	1		< 0.001*			NI
T3/T4	2.254	1.471-3.455				
Lymph node involvement						
Negative	1		< 0.001*			NI
Positive	2.799	1.989-3.938				
TNM stage						
I/II	1		< 0.001*	1		< 0.001*
III	2.319	1.658-3.242		2.150	1.523-3.034	
Smoking						
Never	1		0.004*	1		0.084
Ever	1.683	1.178-2.407		1.445	0.952-2.192	
Alcohol consumption						
Never	1		0.007*	1		0.818
Ever	1.597	1.139-2.240		1.049	0.698-1.577	
mGPS						
0	1		0.042*	1		0.341
1/2	1.514	1.016-2.257		0.760	0.432-1.337	
FC score						
0	1		< 0.001*	1		0.004*
1/2	1.879	1.333-2.648		2.070	1.266-3.385	

OS, overall survival.

* *P* < 0.05.

**Figure 2:**
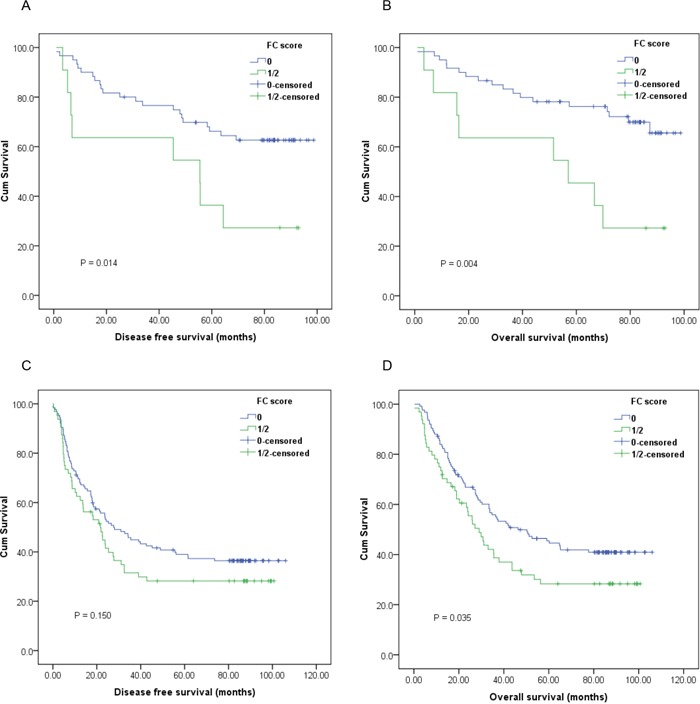
Kaplan-Meier survival curves of **A.**, DFS and **B.**, OS stratified by preoperative FC score in T1-2 stage ESCC patients (n=71); **C.**, DFS and **D.**, OS stratified by preoperative FC score in T3-4 stage ESCC patients (n=189) (with log-rank test).

**Figure 3:**
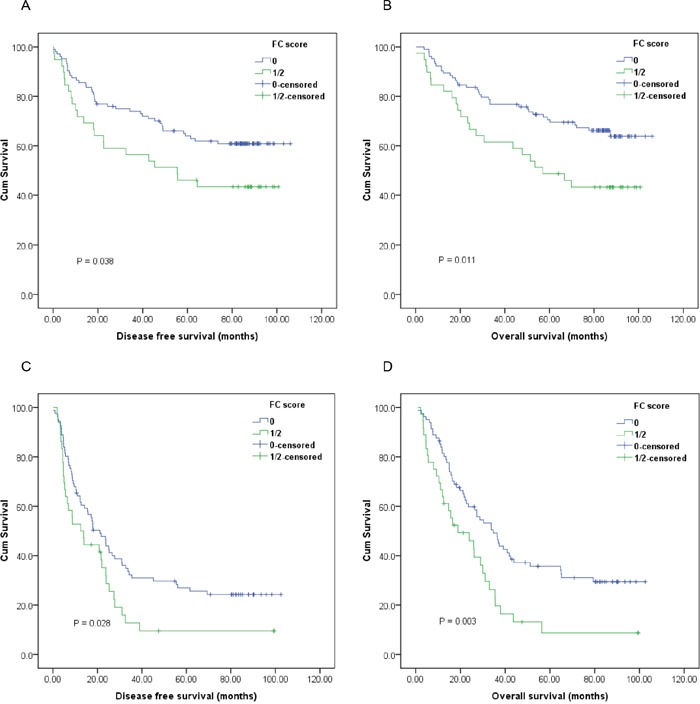
Kaplan-Meier survival curves of **A.**, DFS and **B.**, OS stratified by preoperative FC score in ESCC patients without lymph node involvement (n=143); **C.**, DFS and **D.**, OS stratified by preoperative FC score in ESCC patients with lymph node involvement (n=117) (with log-rank test).

Furthermore, subgroup analysis based on different T stages and lymph node status indicated that high preoperative FC score was significantly correlated with unfavorable DFS (Figure 2A, 3A, 3C; *P*<0.05) and OS (Figure 2B, 2D, 3B, 3D; *P*<0.05) in certain patients, but not DFS in T3-4 stage ESCC patients (Figure 2C; *P*>0.05).

## DISCUSSION

Nowadays, neoadjuvant chemoradiotherapy followed by radical surgery has been recommended as the optimal treatment strategy for locally advanced ESCC. However, to the best of our knowledge, in most less-developed urban and rural areas of China, as well as in low-incoming countries such as African and Central Asia, due to the unreasonable and imbalanced health resources allocation, together with the lack of experienced and professional multidisciplinary treatment team, a vast majority of locally advanced ESCC cases were initially referred to the department of thoracic surgery without preoperative treatment, leaving radical surgery alone or in combination with adjuvant treatment as the “standard care” in such regions.

The present study demonstrated that preoperative FC score was significantly correlated with the clinical stage and postoperative long-term survival, suggesting that those with high preoperative FC scores have more advanced and progressive disease, as well as impaired prognosis. Thus, patients who had high risk of local recurrence or distant metastasis could be identified according to the preoperative FC score, and neoadjuvant induction or further intense adjuvant treatment should be suggested. Additionally, further subgroup analysis revealed that preoperative FC score remained significantly prognostic for both DFS and OS in certain patients, whereas with the exception of DFS in T3-4 stage ESCC patients. In fact, approximately 50% of the two group patients with T3-4 disease experienced postoperative local recurrence or distant metastasis within two years after the surgery. However, the reliable underlying mechanism for elucidating the phenomenon has yet to be investigated. And most of them received salvage treatment, including local radiotherapy and/or systematic chemotherapy thereafter. To the best of our knowledge, this study was the first one reporting the prognostic value of preoperative FC score in ESCC.

mGPS is widely used as a valuable and convenient prognostic indicator in various malignancies, including ESCC [8, 23–25]. However, we failed to identify mGPS as an independent prognostic indicator in the present study. Neither did Arigami et al. found its independent prognostic significance in 238 ESCC patients who underwent esophagectomy with lymphadenectomy [26]. As previous studies have suggested that in operable esophageal cancer patients, systemic inflammation and nutritional deficiencies were insufficient for high-risk stratification [22, 27–28]. Besides, albumin levels were not always decreased in esophageal cancer patients [22]. Therefore, we selected plasma fibrinogen as an alternative.

As an essential hemostatic factor, fibrinogen is synthesized in the liver and secreted into the circulation, and then converted to fibrin in response to infection, tissue injury or inflammation [29–32]. Few studies examining the clinical significance of plasma fibrinogen in esophageal cancer have been conducted. Results from two previous studies indicated that pretreatment plasma fibrinogen was significantly correlated with tumor progression and metastasis in ESCC patients [14], and it could predict postoperative recurrence for those who received neoadjuvant therapy [15]. In addition, Wang et al. found that hyperfibrinogenemia was a valuable predictor for disease progression in resectable ESCC. However, they failed to determine it as an independent prognostic indicator in multivariate analysis [16]. Moreover, Zhang et al. showed that increased plasma fibrinogen level was significantly associated with elevated risk of ESCC, and preoperative hyperfibrinogenemia was a negative prognostic factor for survival of patients with ESCC [17]. Furthermore, as an systematic inflammation response marker, CPR has also been identified as an independent prognostic indicator for various malignancies [20–21]. Therefore, in combination with fibrinogen, CRP might show more potent prognostic value among ESCC patients.

Notably, only five patients were allocated an mGPS of 2 in the present study, leading to an inferior risk classification. As prior studies indicated that systemic inflammation and nutritional deficiencies might not be serious in early or locally advanced esophageal caner patients. Therefore, those with elevated CRP and decreased albumin levels could not be commonly observed [22, 27–28]. Furthermore, the COX multivariate regression analysis identified TNM stage and preoperative FC score as significant prognostic indicators for both DFS and OS, suggesting that preoperative FC score was superior to mGPS in predicting long-term survival in ESCC patient who underwent transthoracic esophagectomy.

Although this study was mainly limited to its retrospective and single-center design, the results showed that preoperative FC score might serve as a new potential marker to predict long-term survival, and facilitate more accurate risk stratification and individualized multidisciplinary treatment for ESCC patients. However, further prospective and large cohort studies are needed to validate these findings.

## PATIENTS AND METHODS

### Patients

A consecutive cohort of 260 patients with histopathologically diagnosed ESCC who underwent curative transthoracic esophagectomy at the Department of Thoracic Surgery, Anhui Cancer Hospital and Anhui Provincial Hospital between January 2007 and December 2011. Patients who received preoperative chemotherapy and/or radiotherapy, patients who had concurrent disease that would affect the hemostatic system (e.g. liver disease and blood coagulation disorders), those who received anticoagulants, corticosteroids, estrogen, or aspirin treatment within 1 month before the surgery, patients who had systemic diseases such as systemic lupus erythematosus (SLE), acquired immunodeficiency syndrome (AIDS), nephrotic syndrome (NS) and rheumatoid arthritis (RA), and those diagnosed with chronic inflammatory diseases or infections, as well as >= grade 2 hypoalbuminemia were also excluded. This study was approved by the independent ethics committees at our hospital and was performed in accordance with the ethical standards of the World Medical Association Declaration of Helsinki.

### Treatment and follow up

All patients underwent a left or right transthoracic esophagectomy with curative intent, and at least a two-field regional lymphadenectomy, including standard, extended, or total dissection of the cervical, thoracic and abdominal lymph nodes, was performed. None of the included patients received adjuvant treatment. Postoperative followed-up including upper gastrointestinal endoscopy, tumor marker and computed tomography were regularly evaluated every 6 months for 5 years after surgery. Disease free survival (DFS) was defined as the date of surgery to local recurrence/distant metastasis or to the last date of follow-up, OS was the time interval from the date of diagnosis to death from any cause or to the most recent follow-up.

### Clinical and laboratory parameters

Patients' clinicopathologic variables, preoperative plasma fibrinogen, serum CRP and albumin levels, as well as postoperative survival were retrospectively reviewed and collected from the medical records. The AJCC/UICC TNM staging system (the 7th edition) was utilized to classify the tumor stage. The tumor length was defined as the long diameter measured with the general post-operative pathological specimens. The degree of differentiation was categorized into poorly/not differentiated, moderately differentiated and well differentiated. And the tumor locations were classified into upper esophagus, middle esophagus and lower esophagus.

Preoperative plasma fibrinogen, serum CRP and albumin levels were examined in samples obtained within 7 days prior to surgery. Plasma fibrinogen concentrations were determined using an automatic coagulation analyzer (Beckman Coulter DC800, USA). Serum CRP and albumin levels were tested by an automatic biochemical analyzer (Roche 501, Japan).

### Fibrinogen and C-reactive protein score (FC score) and modified glasgow prognostic score (mGPS)

The optimal cut-off value for fibrinogen and CRP was defined as 4.0 g/dL and 10.0 mg/L according to previous reports [7–8, 18–20]. Patients with elevated fibrinogen (≥ 4.0g/dL) and CRP (≥ 10.0 mg/L) levels were assigned an FC score of 2, those with only one of these two abnormalities were allocated a score of 1, and those with neither of the two abnormalities were assigned a score of 0.

As previously described, patients with decreased CRP (< 10.0 mg/L) levels were allocated an mGPS of 0, those with both elevated CRP (> 10.0 mg/L) and albumin (> 35.0 g/L) were assigned a score of 1, while patients with both elevated CRP and decreased albumin (< 35.0 g/L) were allocated a score of 2.

### Statistical analysis

Differences between categories were identified using the Chi-square test. Survival curves were estimated using the Kaplan-Meier method, and differences were compared with log-rank test. Univariate and multivariate analysis were performed using Cox proportional hazards regression models and hazard ratios (HRs) for variables respecting to DFS and OS were calculated. HRs with 95% confidence intervals (CIs) and two-sided *P* value were reported. All statistical analyses were performed with SPSS 17.0 (SPSS Inc., Chicago, IL, USA). And a two-sided *P* value of less than 0.05 was considered to be statistically significant.
